# A population of bang-bang switches of defective interfering particles makes within-host dynamics of dengue virus controllable

**DOI:** 10.1371/journal.pcbi.1006668

**Published:** 2019-11-11

**Authors:** Tarunendu Mapder, Sam Clifford, John Aaskov, Kevin Burrage

**Affiliations:** 1 School of Mathematical Sciences, Queensland University of Technology, Brisbane, Queensland, Australia; 2 Australian Research Council Centre of Excellence for Mathematical and Statistical Frontiers, Queensland University of Technology, Brisbane, Queensland, Australia; 3 Centre for Mathematical Modelling of Infectious Diseases, London School of Hygiene and Tropical Medicine, London, United Kingdom; 4 Institute of Health and Biomedical Innovation, Queensland University of Technology, Brisbane, Queensland, Australia; 5 Department of Computer Science, University of Oxford, Oxford, United Kingdom; University of Virginia, UNITED STATES

## Abstract

The titre of virus in a dengue patient and the duration of this viraemia has a profound effect on whether or not a mosquito will become infected when it feeds on the patient and this, in turn, is a key driver of the magnitude of a dengue outbreak. The assessment of the heterogeneity of viral dynamics in dengue-infected patients and its precise treatment are still uncertain. Infection onset, patient physiology and immune response are thought to play major roles in the development of the viral load. Research has explored the interference and spontaneous generation of defective virus particles, but have not examined both the antibody and defective particles during natural infection. We explore the intrinsic variability in the within-host dynamics of viraemias for a population of patients using the method of population of models (POMs). A dataset from 208 patients is used to initially calibrate 20,000 models for the infection kinetics for each of the four dengue virus serotypes. The calibrated POMs suggests that naturally generated defective particles may interfere with the viraemia, but the generated defective virus particles are not adequate to reduce high fever and viraemia duration. The effect of adding excess defective dengue virus interfering particles to patients as a therapeutic is evaluated using the calibrated POMs in a bang-bang (on-off or two-step) optimal control setting. Bang-bang control is a class of binary feedback control that turns either ‘ON’ or ‘OFF’ at different time points, determined by the system feedback. Here, the bang-bang control estimates the mathematically optimal dose and duration of the intervention for each model in the POM set.

## Author summary

Dengue virions with deletions or defects in their genomes can be recovered from dengue patients. These defective viruses can only replicate with the assistance of fully functional viruses and they reduce the yield of the fully functional viruses. They are known as defective interfering (DI) particles. By administering additional, defined, DI particles to patients it may be possible to reduce the titre and duration of their viraemia. This, in turn may reduce the severity of the disease and the likelihood that the dengue virus will be passed from the patient to a mosquito vector. This study estimates the number of DI particles that would need to be administered, and over what period, to have a significant effect on patient viraemia and subsequent dengue fever severity.

## Introduction

Dengue is caused by four serotypes (1-4) of a virus of the same name [1]. The viruses are transmitted between human hosts by Aedes mosquitoes, most commonly *Aedes aegypti*. Almost everyone living between the Tropics of Cancer and Capricorn is at risk of infection and an estimated 300 million infections occur each year [2, 3]. Disease symptoms range from a mild febrile illness to haemorrhagic fever and hypovolemic shock which, if untreated, is fatal in about 30% of cases [4]. Mosquito control programs have had little measurable effect on the number of reported cases of dengue [5], there is no vaccine and no disease specific therapy. Patients are treated by managing the symptoms with which they present.

Infection with one dengue virus (DENV) serotype probably results in life long immunity to re-infection with that DENV serotype but a second infection, with a different serotype, carries a significant risk of developing severe disease [6]. However, the onset of the severe symptoms in secondary infections usually occurs as the viraemia is waning and the secondary immune response is underway [7, 8]. There is a broad correlation between the magnitude of the viraemia in a dengue patient and the severity of the associated symptoms [9]. Any process that reduces the initial viraemia in dengue patients might reduce disease severity and also the risk that a mosquito feeding on the patient would become infected and pass the virus to a new host.

Populations of DENV include virions with genomes with defects ranging from single nucleotide changes [10] to deletion of more than 90 per cent of the genome [11]. Some of these are transmitted in nature for a year or more [10]. DENV virions containing genomes with extensive deletions interfere with the replication of wild type viruses. This phenomenon has been observed with a large number of viruses, mostly with RNA genomes [12, 13]. Furthermore, it has been possible to demonstrate that virions with defective genomes reduce the yield of virus from cells infected with wild type DENV and are known, therefore, as defective interfering (DI) particles [14–16].

There is an extensive literature on the activity of DI particles across a wide range of RNA viruses but interest waned in the 1990s [13, 17]. With the advent of tools to better define DI genomes and to produce artificial ones, there has been a renewed interest in their therapeutic potential and the possibility that they could be used to block transmission of agents such as DENV. However current mathematical models of dengue [18–20] cannot capture all the aspects of virus transmission and no model incorporates defective interfering (DI) particles. A few intracellular, intra-host and population models are available on different infectious diseases such as influenza, scabies, and optimal design for disease control [21–23]. This study uses data from 208 dengue patients in a clinical setting [8] in order to estimate the therapeutic potential of DENV DI particles.

We propose a model inspired by the Clapham *et. al*. [19] and Frank [24] models. The Clapham *et. al*. is an improved extension of their previous model [20] and focused on two models of antibody actions against DENV-1 and DENV-2 infections. In model 1, the antibody kills infected cells through antibody-dependent cell cytotoxicity (ADCC), and in model 2, the antibody assists virus clearance through opsonisation. The authors do not consider the interference of DI particles and both models 1 and 2 do not act simultaneously in the same infection system. The Frank model is a generic model for within-host virus infection kinetics with multiple passages. The main principle of the Frank model is to observe the ‘*von Magnus oscillation*’ in the virus population, occurring due to the existence of DI particles at multiple passages, similar to a ‘predator-pray’ dynamics [25]. The model does not consider any immune response but classifies the infected cells into several categories according to the order of infection by virus particles and, or defective particles as early and late infected cells, co-infected cells and super-infected cells. The Frank model also has a spatial component regulating the density dependent cell division and virus replication. Our model considers the antibody response in viral neutralisation and the natural generation of DI particles. As antibody dependent cell cytotoxicity (ADCC) is not as likely as virus opsonisation, we do not consider ADCC in the present model.

In the present paper we propose that we can account for the inherent variability in the dengue-infected patient data and find a modelling paradigm based on population of models and optimal control that allows us to quantify the effectiveness of DI particles in controlling the viraemia. We build an ensemble, population, of models, in which each element in the population is a mathematical model with exactly the same framework, but where each model has a different set of parameter values for the same set of parameters. All of these parameter values are calibrated against multiple biomarker data. A model is selected in the population of models if its output over a number of febrile days lie in the ranges of the biomarker data. In particular, we calibrate the data for plasma viral load and antibody response for 208 patients in our POMs. Most of the patients have high viraemia amplitudes during the illness. However, the antibody data has been collected on two random days within their febrile periods and that cannot explain the exact dynamics of the antibody response, even asymptotically. In some cases the biomarker data can have extreme values, but we should not and do not ignore these values. Our POMs are not constructed with any guaranteed distribution on the output values of the models. With POMs, we try to explore the range of variability in different cell-virus interactions and the immune responses. There are many different ways of calibrating the POMs [26–28]. In a previous paper [26] we constructed population of models in such way that the distribution of the biomarkers matched as well as the distribution in the data. However, we have based our approach on the first reported [27] that used the range to calibrate the POM.

We develop a population of controls (POCs) to the population of symptomatic patients to attenuate the within-host viraemia level and reduce the days of febrile period. Specifically, bang-bang control is used to determine the minimum dose of DI particles that must be delivered to minimise the height and duration of the viraemia. Although control theory is mainly used in engineering, it has become popular in biology recently. Optimal control of disease treatment, epidemic outbreaks and robust control in protein-protein interaction systems show notable evidences [29–31]. The dynamical programming of optimal control optimises an objective function based on the real time status of the system by invoking the control variable as an external force. Although continuous control has been used in many cases in engineering and biology, bang-bang control is less popular due to possible computer implementational difficulties. Bang-bang control simply flips between the lower and upper boundaries of the control variable as an ‘on-off’ switch depending on the states of the system. It can be viewed as more clinically relevant than continuous control [30, 32]. Recently, we have proposed optimal chemotherapy treatment by continuous and bang-bang control for an acute myeloid leukaemia (AML) model [33].

## Materials and methods

### Within-host viraemia dynamics

To explain the novelty of the present model, we must assert that the competitive dynamics of the DI particles with virus is exhibited in the presence of the antibody response. While the model of Clapham et al. [19] included the role of antibody response in controlling the levels of viraemia, the model assumed that only standard virus is replicated within the host body. Defective interfering particles (DI particles) may also be responsible for the reduction in the production of standard virus [11, 14]. The dynamics of the present model is given by in the following set of ordinary differential equations

$$\begin{array}{c} \begin{array}{cc} \frac{dC_{U}}{dt} & =rC_{U}\left(1-\frac{N}{K}\right)-k\left(V+D\right)C_{U}+\alpha C_{D}\\ \frac{dC_{D}}{dt} & =k\left(C_{U}D-C_{D}V\right)-\alpha C_{D}\\ \frac{dC_{V}}{dt} & =k\left(C_{U}V-C_{V}D\right)-\left(\pi_{1}+\mu \right)C_{V}\\ \frac{dC_{V^{*}}}{dt} & =\pi_{1}C_{V}-\delta C_{V^{*}}\\ \frac{dC_{VD}}{dt} & =k\left(C_{V}D+C_{D}V\right)+\mu C_{V}-\delta C_{VD}\\ \frac{dV}{dt} & =\beta \pi_{2}C_{V^{*}}-\rho V-\epsilon ZV\\ \frac{dD}{dt} & =\gamma \phi C_{VD}-\rho D-\underset{\text{antibody}\;\text{mediated}\;\text{DIP}\;\text{clearance}}{\underset{︸}{\epsilon ZD}}\\ \frac{dZ}{dt} & =\eta_{1}Z\frac{V}{\eta_{2}+V}+\underset{\text{DIP}\;\text{particle-triggered}\;\text{immune}\;\text{response}}{\underset{︸}{\eta_{1}Z\frac{D}{\eta_{2}+D}}}\\ N & =C_{U}+C_{D}+C_{V}+C_{V^{*}}+C_{VD}. \end{array} \end{array}$$ (1)

**Fig 1:**
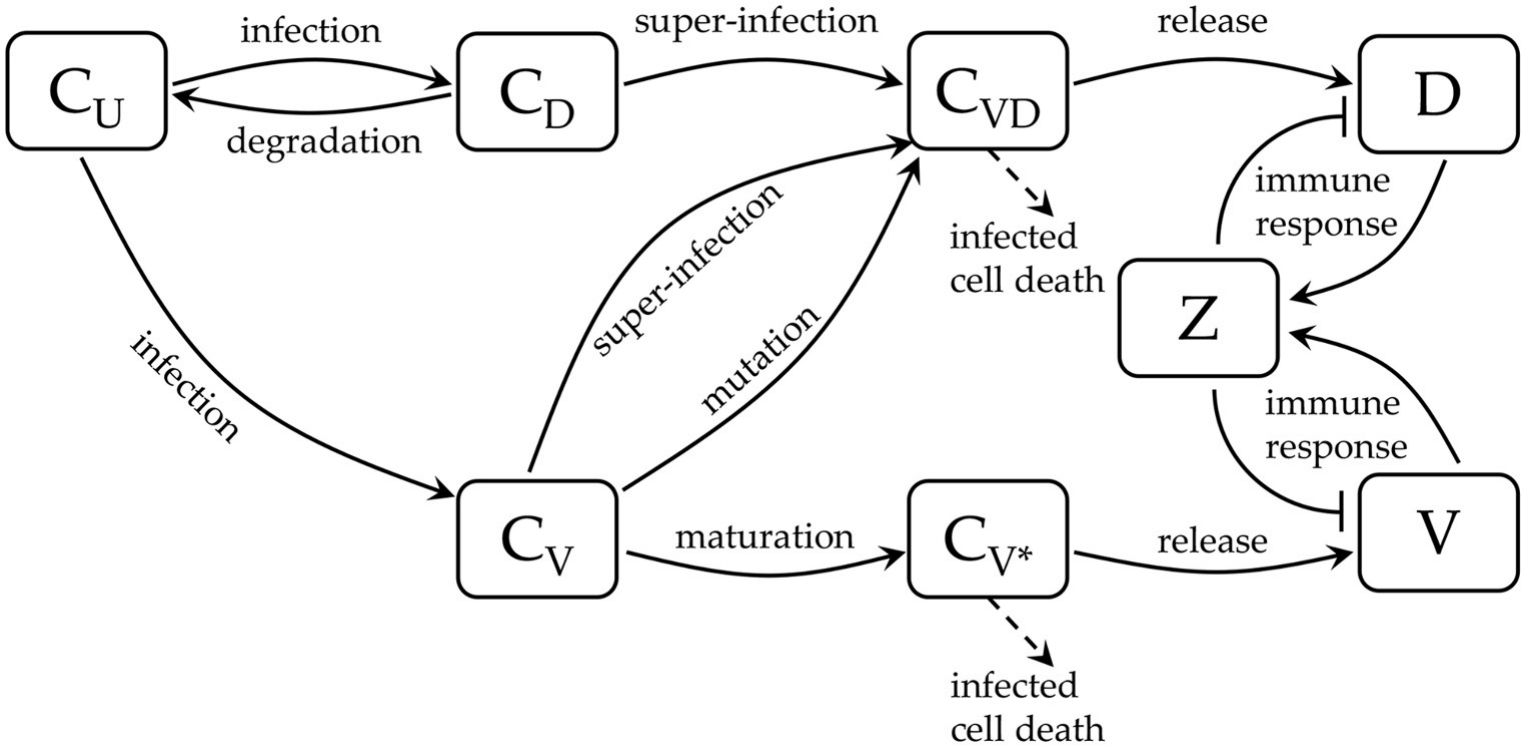
Schematic diagram of the within-host dengue virus infection dynamics. The uninfected target cells (*C*_*U*_) can be infected by either virus particles (*V*), or defective particles (*D*) to generate early infected *C*_*V*_ and *C*_*D*_ cells. The *C*_*D*_ cells can either be transformed into *C*_*VD*_ cells by super-infection, or come back to uninfected state (*C*_*U*_) by losing the DI particles. *C*_*V*_ cells can also be transformed into *C*_*VD*_ cells either being infected by DI particles or through mutation of the virus genomes inside it. Otherwise, *C*_*V*_ cells mature to late infected *C*_V*_ cells. The *C*_*VD*_ and *C*_V*_ cells are able to replicate and release the defective particles and the standard dengue virus, respectively. The occurrence of viral particles of any kind (*V* and/or *D*) in the blood plasma triggers the antibody response (*Z*), which in turn prevents the infected cells (*C*_*VD*_ and *C*_V*_) at virus production. The cell death has been considered only for *C*_*VD*_ and *C*_V*_ infected cells as the other two infected cells (*C*_*D*_ and *C*_*V*_) transform themselves quickly into other states.

With very specific aspects of dengue infection from previous models [17, 19, 24], this new model describes the dynamics of standard virus (*V*) and DI particles (*D*) within the host. We consider the antibody response (*Z*) by the infected cells in virus neutralisation and DI particle clearance. The uninfected target cells (*C*_*U*_) become infected and consequently produce four types of infected cells: infected by DI particles only (*C*_*D*_), virus only (*C*_*V*_), virus-infected and late enough for further infection (*C*_V*_), and infected by both (*C*_*VD*_) (Fig 1).

The assumptions that underpin our new model are described here. Bursting and cell lysis do not occur during the release of dengue virus particles. Infected cells are categorised in two classes according to their stages of infection: early and late. The early infected cells (*C*_*D*_ and *C*_*V*_) are available for super-infection, but the late infected cells (*C*_V*_ and *C*_*VD*_) are not because of the triggered interferon response and alteration in cell membrane receptor dynamics [34, 35]. Once the infected cells start replicating the virus and, or DI particles, the interferon pathway is triggered to destroy the uptaken virus genomes inside the cells and also secrets interferon in the immediate neighborhood. The cell surface receptors (toll-like receptors, etc.) change conformation in the extracellular and cytosolic domains in response to the activation of interferon pathway so that the infecting virus particles cannot dock on the cell surface. Eventually, the late infected cells (*C*_*VD*_, *C*_V*_) die naturally, whereas the early infected cells (*C*_*D*_, *C*_*V*_) transform into the late infection state before natural death as the rate of infection is much faster than the rate of infected cell death [24]. As incorporating natural death terms for the early infected cells cannot contribute significantly to the model output dynamics, we do not consider them in the present model (See S1 Fig). The immune response is strong in the case of secondary infection leading to antibody-dependent enhancement (ADE) of the viraemia while it is very weak in case of primary infection. As the antibody production occurs in a B cell maturation-mediated process, the functional form of the immune response should implicitly take care of the immune cell proliferation [36]. We consider the immune response in a simplified Hill-type function without any cooperativity so that the response is prominent only in the presence of significant antibody level, preferably in case of secondary infection. Both the defective and standard virus particles in this model are equally efficient in the competition of infection or replication and respond in similar way to the antibody, as we do not consider the nucleotide length dependent intracellular replication or packaging kinetics here. Rather, we consider detailed replication and packaging mechanisms in a separate article, where a single cell model explains the replication dependent on nucleotide-length, RNA secondary structure, and diffusion-mediated queuing for RNA encapsidation.

Most of the model parameters must be estimated from the reported base values as the model is quite different from previous models, although the range of their values from the aforesaid papers [19, 24] are informative in creating the population of models. The initial conditions of *C*_*U*_, *C*_*D*_, *C*_*V*_, *C*_V_*, *C*_*VD*_ and *D* are considered constant at the start of infection. Only the initial viral load (*V*_0_) and antibody levels (*Z*_0_) for each patient have been sampled in the population of models. The patient-specific parameters (*α*, *δ*, *η*_1_, *η*_2_, *π*_1_, *π*_2_, *ϕ*) are sampled using Latin Hypercube sampling (LHS) within the physiological range. LHS is a way of sampling high dimensional parameter spaces so that the number of samples does not scale with the dimension [37]. The way this is done is to discretise a *d* dimensional parameter space with some mesh and then place a cross in a box such that there is only ever one cross in each *d* − 1 dimensional subspace. A cross means that box is sampled at random for the *d* parameter values. The remaining parameters have been classified into two classes: natural human host parameters (*r* and *K*), which are constant in the complete set of POMs, and serotype-specific parameters (*β*, *ϵ*, *γ*, *k*, *μ*, *ρ*), which stay constant for a POMs of a particular serotype. We tabulate the description of the rate parameters in Table 1.

**Table 1:** Kinetic rate parameters and their sampling ranges used in the POMs.

Parameters	Values	Descriptions	Source
Natural human host parameters			
K	3.505 ×10^7^ cells per ml	Cellular carrying capacity of proliferation	[24]
r	15.217 per day	Intrinsic rate of host cell proliferation	[24]
*C*_*U*0_	1.0 ×10^8^ per ml	Level of uninfected target cells on the day 0 of illness	-
Serotype-specific parameters			
*β*	758.045	Number of *V* released per *C*_V*_ cells after packaging	[24]
*ϵ*	16.225 per day	Antibody-mediated virus neutralisation	[19]
*γ*	38.259	Number of *D* released per *C*_*VD*_ cells after packaging	[24]
k	2.45 ×10^−10^ per day	Rate of infection per virus	[19]
*μ*	37.651 per day	Mutation rate of *V* to *D* within host cells, turning *C*_*V*_ cells into *C*_*VD*_ cells	[24]
*ρ*	9.562 per day	Natural clearance rate of *V* and *D*	[19]
Patient-specific parameters			
*α*	5.836 ×10^±2^ per day	Rate of loss of DI particles within host cells, turning *C*_*D*_ cells into *C*_*U*_ cells	[24]
*δ*	2.426 ×10^±2^ per day	Death rate of infected cells	[19]
*η*_1_	1.607 ×10^±2^ per day	Proliferation rate of triggered immune response per infected cells by *V* or *D*	[19]
*η*_2_	2.0 ×10^8±2^	Threshold parameter of the triggered immune cells proliferation	[19]
*π*_1_	9.863 ×10^±2^ per day	Rate of maturation of *C*_*V*_ cells into *C*_V*_ cells	[24]
*π*_2_	68.503 ×10^±2^ per day	Rate at which each *C*_V*_ cells produces *V*	[24]
*ϕ*	21.782 ×10^±2^ per day	Rate at which each *C*_*VD*_ cells produces *D*	[24]
*V*_0_	3.6 ×10^5±2^ per ml	viraemia level on the day 0 of illness	-
*Z*_0_	5.645 ×10^−2±2^ per ml	Level of immune response on the day 0 of illness	-

### Population of models

Variability inherently occurs in many biological and physiological measurements and we cannot avoid them. Every patient, for example, may have very different responses to an infection or a treatment and we need to account for this variability. Sometimes we aggregate the data and fit the model to the mean trajectory or choose a subset of the data as being representative or the hypothetically best sets of data and extrapolate those features to the large population. This can reduce the errors in measurement, but is unable to capture the intrinsic variability in the system. Hence, analysing models in a population from a set of measured data and exploring the hidden features intrinsic to the system is more effective for predicting physiological phenomena when there is inherent variability.

As our model is based on a consolidation of two different models, an initial estimation of the model parameters is essential. We use ‘arFitLHS’ tool of ‘Data2Dynamics’ package in Matlab for initial parameter estimation for the base model [38]. For this parameter estimation, we use the median of the viraemia and antibody response data for each serotype. We generate multiple candidate models with parameters sampled by Latin Hypercube Sampling. We are at liberty to choose different criteria for our calibration. Previous work has calibrated to the ranges of the data [28], but this is somewhat crude. More recently, we proposed calibration based on matching the distributions in the data available [26]. This means that appropriate outputs from the POM matches the data in a distribution setting. In the present article we are following the earlier approach. First, Latin Hypercube sampling is performed to generate 20,000 parameter sets for 7 patient-specific parameters (*α*, *δ*, *η*_1_, *η*_2_, *π*_1_, *π*_2_, *ϕ*) and initial conditions of virus (*V*_0_) and antibody response (*Z*_0_) with the serotype-specific parameters (*β*, *ϵ*, *γ*, *k*, *μ*, *ρ*) constant for each serotype, simultaneously. We keep the natural human host parameters (*r* and *K*) same for the four serotypes. The parameter sets generated in LHS are used to simulate 20,000 variants of the same model. Hence, we generate a very large initial population of models (20,000) for each serotype. The model calibration is the next step that decides whether a model should be included or not in the final POMs. We use upper and lower values of the available biomarker data on each day of illness as the allowed range of acceptance for the model output variability. We select only those models that cover the range for the biomarker results on each day of illness. This calibrated population possesses all plausible models with dynamic variability within the data range.

### Optimal bang-bang control

The aim of optimal control is to determine the temporal profile of a control variable that optimises a defined objective function. The objective function, or the payoff, is structured from the state and control variables along the time trajectory and/or at the final time. There are two ways of implementing optimal control. One is continuous and differentiable. The other one is continuous but occurs as a step function and is known as bang-bang control, in which the control is either on or off. In practical settings bang-bang control is more appropriate for intervention and that is what we use here.

We follow the algorithmic steps for optimal control for a nonlinear system of ODEs as follows

1. Describe the system with the control variable (**u**) and initial state, **x**(0) = **x**_0_ as
  $$\frac{dx}{dt}=A\left(x,t\right)x+B\left(x,t\right)u+C.$$ (2)
  The final time *T*_*f*_ and final state **x**(*T*_*f*_) should be specified as free or fixed according to the context of the problem. In the present model, we use fixed final time *T*_*f*_ and free final state **x**(*T*_*f*_).
2. Construct the payoff functional in terms of running cost (*L*) and terminal cost (*ϕ*) functional as
  $$\underset{u\epsilon [0,u_{b}]}{\text{min}}(J\left(.\right)≔\phi \left(x\left(T_{f}\right),T_{f}\right)+\int_{T_{0}}^{T_{f}}L\left(x,u,t\right)dt),$$ (3)
  where **u** is the control variable, or vector of control variables, with bounds **0** ≤ **u** ≤ **u**_*b*_. By choosing an optimal control u*(*t*) and solving the state **x**(*t*), one can find the optimal payoff. The optimal control can be determined by solving the necessary conditions through Pontryagin’s minimum principal (PMP) [39, 40].
3. Construct the Hamiltonian following PMP for an unconstrained problem
  $$H=\lambda^{T}\left(A\left(x,t\right)x+B\left(x,t\right)u+C\right)+L\left(x,u,t\right).$$ (4)
  For bang-bang control, *L*(**x**, **u**, *t*) can be written in a linear form as *L*_1_(**x**, *t*)**x** + *L*_2_(**x**, *t*)**u** + *L*_3_ and the Hamiltonian must be rewritten in the form
  $$H=\lambda^{T}\left(A_{1}\left(x,t\right)x+B_{1}\left(x,t\right)u+C_{1}\right),$$ (5)
  where, **A**_1_ = **A** + (1/λ)**L**_1_(**x**, *t*), **B**_1_ = **B** + (1/λ)**L**_2_(**x**, *t*) and **C**_1_ = **C** + (1/λ)**L**_3_. Here the lambda are the elements of the vector of Lagrange multipliers or the adjoint variables for an unconstrained control problem. Negative partial differential of the Hamiltonian with respect to each state variable (*x*_*i*_, *i* = 1, 2, …) generates corresponding costate equation, which is the time derivative of the adjoint variable (λ_*i*_, *i* = 1, 2, …) as
  $$\frac{\partial \lambda_{i}}{\partial t}=-\frac{\partial H}{\partial x_{i}}.$$ (6)
  In the present model, we have eight costate equations corresponding to eight state equations (Eq 1).
4. From the Pontryagin’s minimum principal, the switching function for a bang-bang control is
  $$\frac{\partial H}{\partial u}=\lambda^{T}B_{1}\left(x,t\right).$$ (7)
  The values of the switching function can be positive or negative. A zero value of the switching function represents singular control. The particular time points, where the switching function changes sign are known as the switching points and the duration between the switches are called the bang times (*τ*’s). After every bang time (*τ*_1_, *τ*_2_,‥), the bang-bang control variable turns on or off depending on the direction of switching.
5. The optimal bang-bang control (u*(*t*)) flips between the bounds, [0, **u**_*b*_] at the switching points as
  $$u^{*}\left(t\right)=-sign\left(\lambda^{T}B_{1}\left(x,t\right)\right)u_{b}.$$ (8)
  In the present study we use one control variable (*u*(*t*)), the administration of excess DI particles to the model to reduce the viral infection as well as quick clearance of the virus from the host. For the present POMs of four dengue serotypes, the range of the viraemia growth is large (approximately 10^3^ to 10^11^). For that reason it is difficult to decide on upper bounds of the control (*u*_*b*_) for these POMs. We determine the *u*_*b*_ from the individual uncontrolled viraemia profile for each model considered to be controlled.

In dynamical programming of bang-bang control for linear systems, the control can be computed numerically using boundary value problem (BVP) solvers [41]. But a nonlinear two-point boundary value problem (TPBVP), such as our present model, cannot be solved directly with traditional numerical boundary value problem solvers. We use the forward-backward sweep method, where ordinary differential equations solvers are used twice: forwards for the state equations and backwards for the costate equations [42]. Then we update the switching function(∂*H*/∂*u*) and the control (*u*(*t*)) [30, 43]. We use Pontryagin’s minimum principle and solve the discontinuous right hand side of the state and co-state equations. We note that this method needs many more iterations than continuous control methods to converge. However, for models with strong non-linearity such as stiff and oscillatory control problems, this approach is reasonably efficient.

#### Control strategy for dengue fever

As our aim is to control dengue within host, we construct an objective function in terms of the running cost functional only and do not include a terminal cost (see Eq 3). The reason for this is that the infection and virus are naturally cleared at the final time point and so terminal cost functional is insignificant in such cases. Thus we take

$$\underset{u\epsilon [0,u_{b}]}{\text{min}}(J\left(.\right)≔\int_{T_{0}}^{T_{f}}(\frac{1}{2}aV^{2}\left(t\right)+\frac{1}{2}bC_{V}^{2}\left(t\right)+cu\left(t\right))dt),$$ (9)

where *T*_0_ and *T*_*f*_ are the initial and final time, and *a*, *b* and *c* are constants to be determined from the optimal control problem. Note that the squared terms ($\frac{1}{2}V^{2}\left(t\right)$ and $\frac{1}{2}C_{V}^{2}\left(t\right)$) act like an energy in the system (a Hamiltonian) while the linear term in the control implies that we use bang-bang (on-off) control rather than a continuous control. We assume equal weighting on the three terms, so *a* = *b* = *c* = 1. In the course of dengue control, we prefer to apply a bang-bang control rather than a continuous control. Here, the administration dose rate (*u*(*t*)) of DI particles is the control variable. The medical nomenclature of the purified DI particles is therapeutic interfering particles or TIPs. In order to make the vaccination program cost-effective and reduce the time course of the vaccination process this information is included in the structure of the payoff function during the optimisation. As the plasma viraemia (*V*) and the cellular infection of all kinds (*C*_*V*_, *C*_V*_) show a rapid growth in the first 2-4 days of the febrile period and are cleared within 10-12 days, we seek to minimise the peak of the viraemia (*V*) and virus-infected cells (*C*_*V*_) that in consequence may help reduce all the infections. The DI particles within the host (*D*) compete with the virus for the uninfected cells (*C*_*U*_) and that is an advantage to introduce a large number of DI particles to inhibit the viral infection. The system of ordinary differential equations (Eq 1) can be rewritten after introducing the control variable, *u*(*t*) as

$$\begin{array}{c} \begin{array}{cc} \frac{dC_{U}}{dt} & =rC_{U}\left(1-\frac{N}{K}\right)-k\left(V+D\right)C_{U}+\alpha C_{D}\\ \frac{dC_{D}}{dt} & =k\left(C_{U}D-C_{D}V\right)-\alpha C_{D}\\ \frac{dC_{V}}{dt} & =k\left(C_{U}V-C_{V}D\right)-\left(\pi_{1}+\mu \right)C_{V}\\ \frac{dC_{V^{*}}}{dt} & =\pi_{1}C_{V}-\delta C_{V^{*}}\\ \frac{dC_{VD}}{dt} & =k\left(C_{V}D+C_{D}V\right)+\mu C_{V}-\delta C_{VD}\\ \frac{dV}{dt} & =\beta \pi_{2}C_{V^{*}}-\left(\rho +\epsilon Z\right)V\\ \frac{dD}{dt} & =u\left(t\right)+\gamma \phi C_{VD}-\left(\rho +\epsilon Z\right)D\\ \frac{dZ}{dt} & =\eta_{1}Z\left(\frac{V}{\left(\eta_{2}+V\right)}+\frac{D}{\left(\eta_{2}+D\right)}\right)\\ N & =C_{U}+C_{D}+C_{V}+C_{V^{*}}+C_{VD}. \end{array} \end{array}$$ (10)

We assign bang-bang controls to the models from the POMs discussed above and obtain a population of controls (POCs) defined by the vectors of the amplitude of the bang of DI administration dose (*u*(*t*)) and on-off time duration (*τ*’s) of the bang-bang switches for each of the four serotypes.

## Results

Our main assumption for this study is that we can leverage existing data sets and mature models to explore the underlying heterogeneity implicit in the data and the processes that are being analysed. We do this using population of models (POMs) of within-host virus dynamics for each of the four dengue serotypes. We generate an initial database of 20,000 candidate models with the parameters chosen through Latin Hypercube Sampling and select only those models for the POMs that generate outputs that lie within the biomarker data range. The effect of virus infection on host innate immunity is recognised indirectly by immune cell proliferation and antibody production. Once the POMs have been calibrated, excess DI particles are used via optimal bang-bang control to inhibit the within-host viral burden and reduce the fever duration.

### Population of models

**Fig 2:**
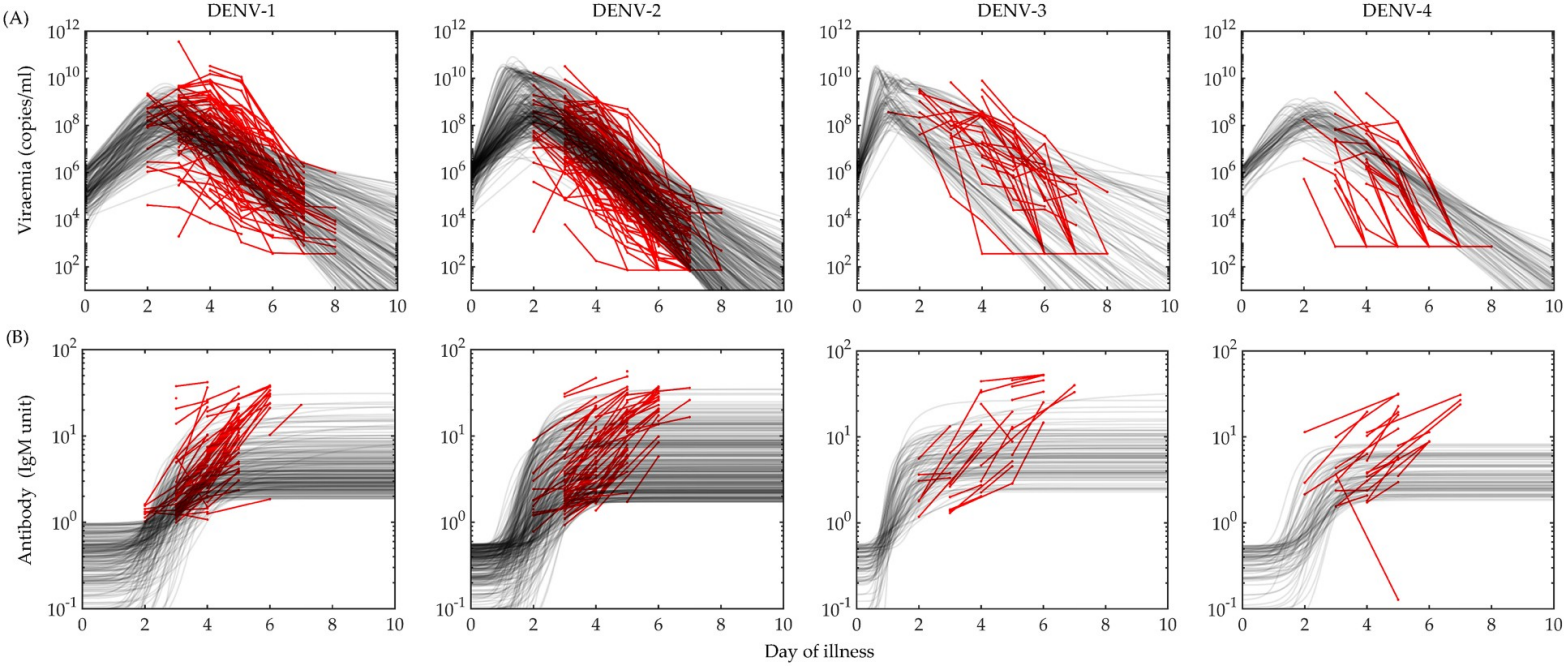
Population of models. The data of (A) viraemia and (B) antibody response for 208 (78 for DENV-1, 83 for DENV-2, 25 for DENV-3 and 22 for DENV-4) hospitalised dengue patients reported in Nguyen *et al* [8] are calibrated to construct serotype specific population of models (POMs). The biomarker data are shown in red lines with dots and the calibrated models from the POMs are plotted in transparent black lines. The calibration is performed for the ranges of available data on each day of illness. The total number of calibrated models in the POMs is 701 (221 for DENV-1, 306 for DENV-2, 93 for DENV-3 and 81 for DENV-4). The level of viraemia on day zero of illness is covered with the heterogeneity generated by the viral load on the day of infection and the incubation period.

From the clinical data, we have a set of 208 adult dengue patients with more than 3 days of fever [8]. Among them, 38% and 40% of cases are DENV-1 and DENV-2 infections and a very low number of cases from DENV-3 (12%) and DENV-4 (11%). Most of the patients enrolled into hospital on days 2, 3 and 4 of their illness with high viraemia load in their blood samples. To build a model with an estimate of the day of infection using the day of illness is not appropriate. The days between the infection and start of illness are known as the incubation period for the plasma viraemia. For a large population of patients, it is difficult to frame the range of this time period in a dynamical model. To address this problem, we consider that the start of illness is a day in between the day of infection and maximum plasma viral load. The fever starts with a range of detectable viraemia load (*V*_0_) on the day the illness starts. Although DI particles are not observed directly in any prior study of blood viraemia trajectories, they are known to occur naturally in viral infection systems. We may predict that effect from our POMs construction, since they are generated naturally in viral infection systems. Fig 2 represents the calibrated POMs (black transparent lines) with the reported plasma viraemia (red lines with dots) for each of the four DENV serotypes for 10 days of their febrile periods. In the initial calibrated POMs, we found many viraemia models with large oscillations and abrupt growth in the antibody models. Although they satisfy the calibration criteria to be included in the final POMs, they are omitted from the analysis as we cannot find any oscillatory behaviour in the reported viraemia data.

**Fig 3:**
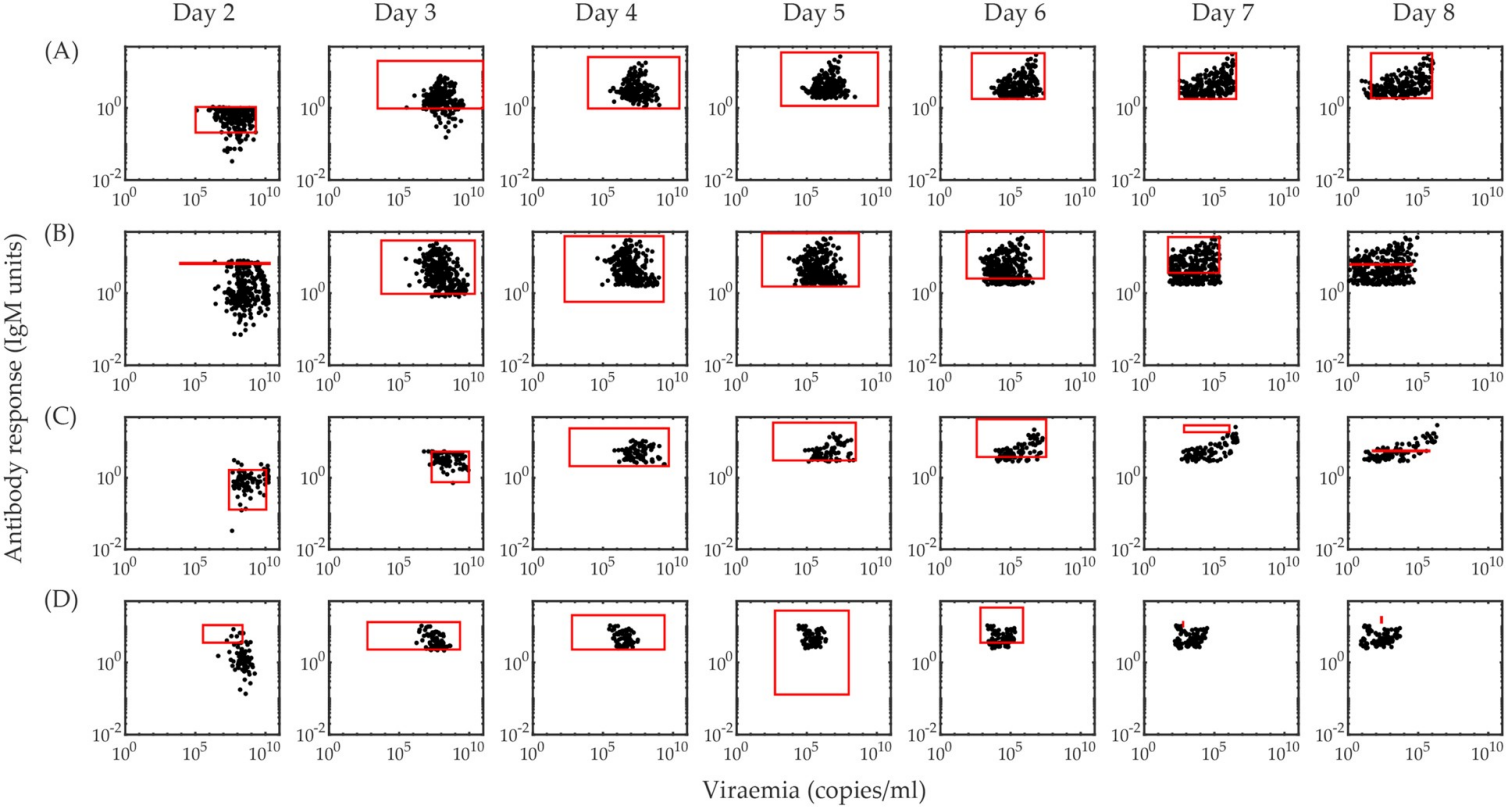
Calibration of the population of models. The scattered plots of antibody response vs. viraemia calculated from the calibrated POMs are shown on each day of illness. Each row represents a serotype as (A) DENV-1, (B)DENV-2, (C) DENV-3 and (D) DENV-4. The red boxes in all plots represent the range of the biomarker levels in the dataset on the particular day of illness. Every scattered plot represents the compact calibration of the models with available bimarker data. We do not show days 1, 9 and 10 as there is no available data for these days. The calibration of DENV-2, DENV-3 and DENV-4 POMs are weakly calibrated on day 2, 7, and 8 due to lack of availability of data. Day 2 and day 8 in the DENV-2 POMs, and day 8 in the DENV-3 POMs do not have any antibody data. In the DENV-4 POMs, day 7 and day 8 have single observations for viraemia and no antibody response data is recorded.

In Fig 2, we present the POMs constructed (black transparent lines) based on the available biomarker data (red lines with dots). The data for the viraemia are regularly collected for every patients from day 2 to day 8 and that is reflected in the calibrated POMs nicely. But the available data for the antibody response is not as consistent as they appear randomly on any two of the days of illness. Calibration of the POMs for these data does not perform as effectively as for the viraemia population. To analyse the POMs for the four serotypes comparatively, we see that the POMs for DENV-2 is the most tightly calibrated with the biomarker data. The POMs for DENV-1 and DENV-4 are well calibrated in the dense region of the data and very few outlying data points cannot be captured in the POMs while DENV-3 POMs captures the spread of the data at every day of illness. In the case of DENV-2 and DENV-3, the recurrence of tiny oscillations near the peaks of their rapid growths in the viraemia are more prominent than in DENV-1 and DENV-4 although that does not affect the antibody response. The antibody dynamics for the four serotypes are quite similar except in DENV-4. It is quite low in comparison to the other serotypes. The coverage of the data spread by the calibrated POMs and the goodness of the calibration is presented by scattered plots in Fig 3.

In Fig 3, we depict the antibody response with respect to corresponding viraemia levels on every day of illness for further clarification of the calibration process. The black dots are the antibody-viraemia data points calculated from the accepted POMs on each day of illness. We show that most of the POMs results stay within the ranges (red boxes) of the biomarker data on day 3, 4, 5 and 6 for all the four serotypes. On days 2, 7 and 8, due to very low number of data-points, the range of detection is not a reliable indicator of goodness of fit for the POMs. If we look at the day-wise calibration of each serotype, the best calibration is observed in case of DENV-1. On days 2 and 8 for DENV-2, there is no available scope for calibration because only a single data point is available for antibody response. A similar situation is observed on days 7 and 8 for DENV-3 and DENV-4.

**Fig 4:**
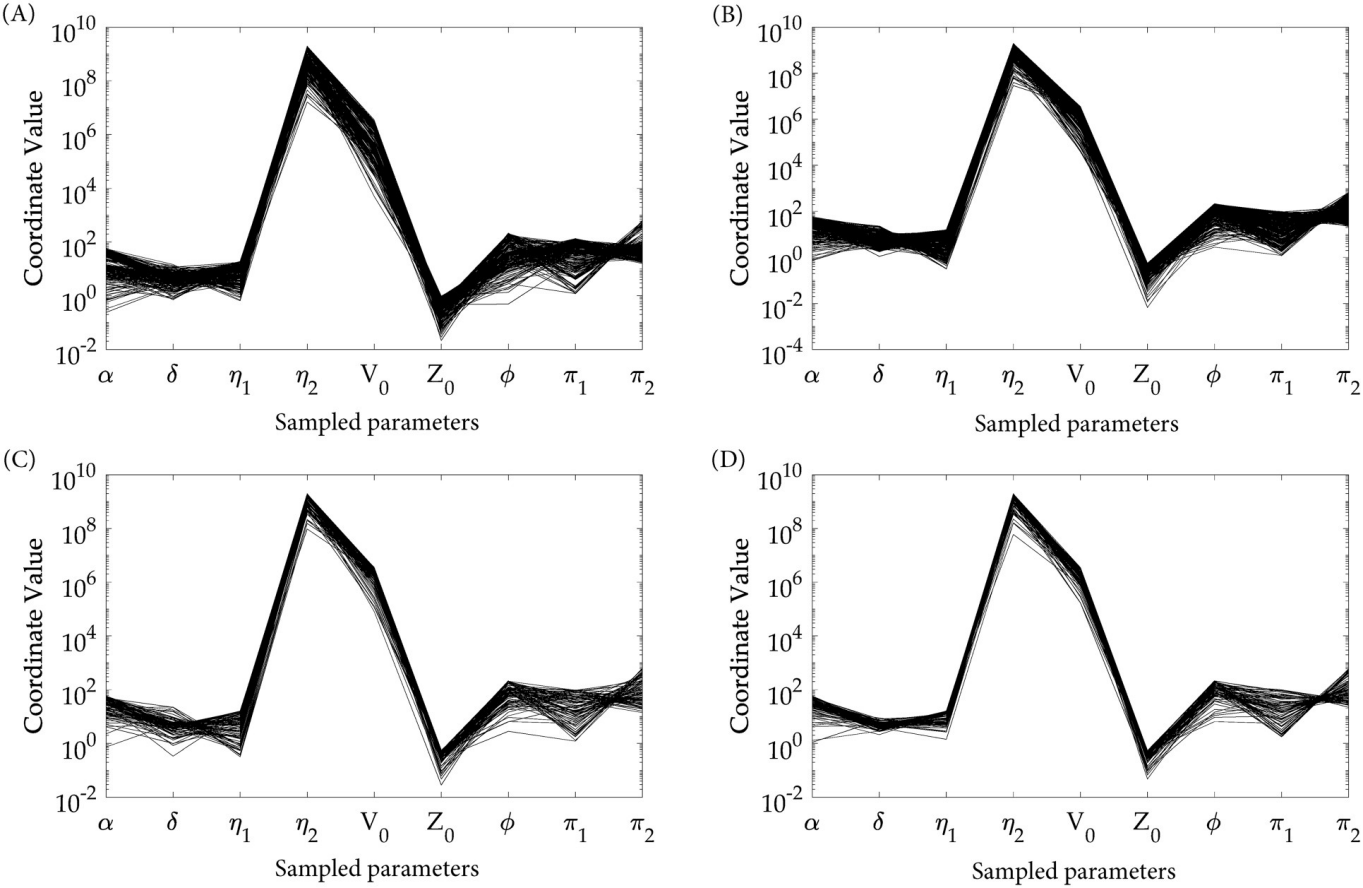
Variability in model parameters. The patient-specific parameters are shown in parallel co-ordinates for the four calibrated POMs of (A) DENV-1, (B) DENV-2, (C) DENV-3 and (D) DENV-4. These parameters values represent the models included in the four serotype-specific POMs. The parameters have been sampled for 20,000 models using Latin Hypercube Sampling from a domain of 10^−2^ to 10^2^ times the initial parameter values and parameter sets for the qualified models included in the parallel co-ordinates. If we draw multiple copies of the y-axis, perpendicular to the x-axis and equidistant with each other, then these represent the axes of the multi-dimensional parallel coordinates for a high dimensional Euclidean system [44, 45]. Any data point in a multi-dimensional space can be mapped on a polyline that connects each axis of the parallel coordinates at a distance proportional to its coordinate value.

The spreads in different patient-specific parameters for the four serotypes are shown in parallel coordinate planner graphs in Fig 4. We consider 7 patient-specific parameters (*α*, *δ*, *η*_1_, *η*_2_, *ϕ*, *π*_1_, *π*_2_) and initial conditions of virus (*V*_0_) and antibody response (*Z*_0_) as the y-axes of the 9-dimensional parallel coordinates. The rate of triggered immune response proliferation (*η*_1_) has notable differences in the case of DENV-4 from the other three serotypes. The effect of this narrow spread in *η*_1_ is reflected in the POMs for the DENV-4 antibody response in Fig 2B. The initial viraemia level (*V*_0_) spreads in a narrow domain for DENV-4 compared with the others and it makes the viraemia POMs in Fig 2A narrow. DENV-3, with its very narrow spread in *V*_0_, appears to be wide in the course of time. In all the cases, the low value of *η*_2_, the threshold of immune response proliferation, is inversely related to high level of *V*_0_.

**Fig 5:**
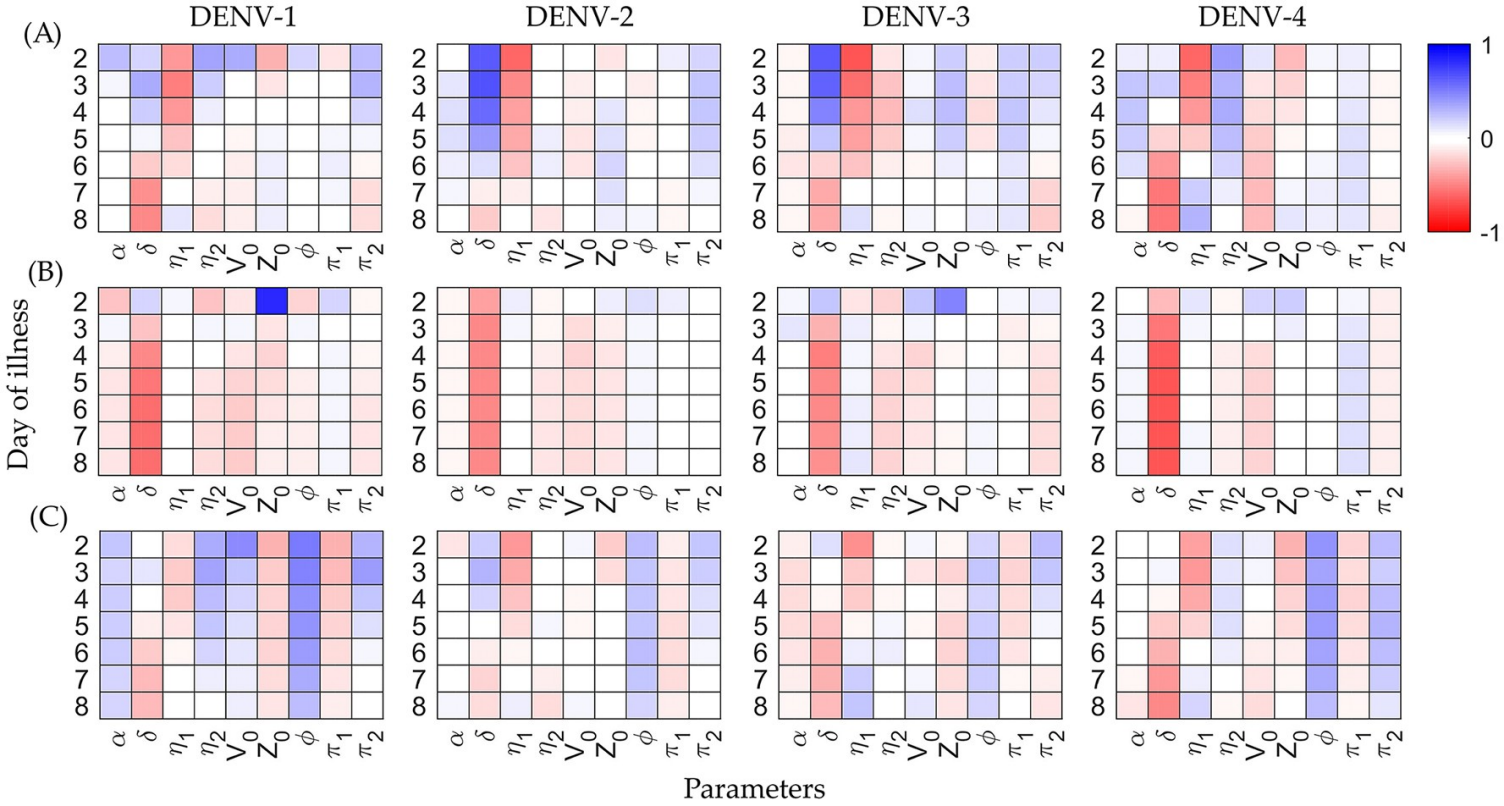
Partial correlation coefficient heatmaps. The partial correlation coefficients are calculated between the patient-specific parameter (*α*, *δ*, *η*_1_, *η*_2_, *V*_0_, *Z*_0_, *ϕ*, *π*_1_, *π*_2_) values and the model outputs (A) viraemia, (B) antibody response and (C) defective particles, respectively in the calibrated POMs. The correlation heatmaps are shown on each day of illness (2-8). The red and blue colours represent negative and positive correlations, respectively while white colour stands for weak or no correlation.

For each of the patient-specific parameters, which have been allowed to vary in the population, the partial correlation coefficient (PCC) is calculated pairwise with the viraemia (*V*), defective particles (D) and antibody response (Z), calculated from the POMs. This correlation based approach can explore the sensitivity of the model parameters in association with the parameter variability. PCC is a parametric measure of sensitivity analysis that detects the degree of association between output and input variables of a dynamic model by removing the existing correlations of the other model variables with these two variables [46, 47]. To calculate PCC for a set of multiple variables, one has to compute the co-factor matrix (C) of the Pearson’s correlation matrix for the variables. The PCC of a pair of variables is defined as $-C_{ij}/\sqrt{C_{ii}C_{jj}}$. Here, PCC identifies one-to-one correlation between a particular model parameter with the specific model output after removing the contributions of all the other model components. Thus it magnifies the one-to-one correlation between the parameter-output pairs. In Fig 5, we present three different heatmaps to quantitatively compare the PCC levels among the patient-specific parameters and the viraemia load, antibody response and accumulated DI particles levels across the four serotypes. Interestingly, although the POMs for viraemia load and antibody response show similar trends, the relation is not just straightforward if we look at the contributions of the model parameters through their PCC values.

In row Fig 5A, the PCCs of viraemia with different parameters are plotted. The death rate of infected cells (*δ*) shows a transition from highly positive to highly negative correlation as long as the illness continues, while the proliferation rate of the triggered immune response (*η*_1_) moves in the opposite direction. However, the threshold parameter of the immune response (*η*_2_) is not following a similar trend across the serotypes. To classify the PCCs for *η*_2_, DENV-1 and DENV-3 are separable from the class of DENV-2 and DENV-4. On the other hand, the rate of DI particle loss (*α*) from *C*_*D*_ cells and production (*ϕ*) by *C*_*VD*_ cells, the rate of *C*_*V*_ cells maturation (*π*_1_) and virus release (*π*_2_), initial condition of viraemia (*V*_0_) and antibody response (*Z*_0_) remain almost in the weak correlation regime with the viraemia for all the serotypes. In row Fig 5B, the PCCs of the antibody response with *δ* show high negative correlation while the rest of the parameters have no significant contributions. In the case of DI particles in row Fig 5C, all the parameters except the production rates for DI (*ϕ*) and virus (*π*_2_) appear with the same trend in Fig 5A, while *ϕ* and *π*_2_ show high positive correlation in all the serotypes on nearly every day of illness.

### Efficacy of DI particle-mediated treatment

Administration of excess DI particles into the DENV-infected host system must have an effect but its efficacy is highly dependent on the day of intervention and the dose of treatment. To observe these two points, we consider a model arbitrarily chosen from the sets of POMs and consider two different strategies: single-time doses of increasing strengths are applied on different days of illness, and bang-bang optimal control is performed for increasing doses that producing optimum payoff values.

**Fig 6:**
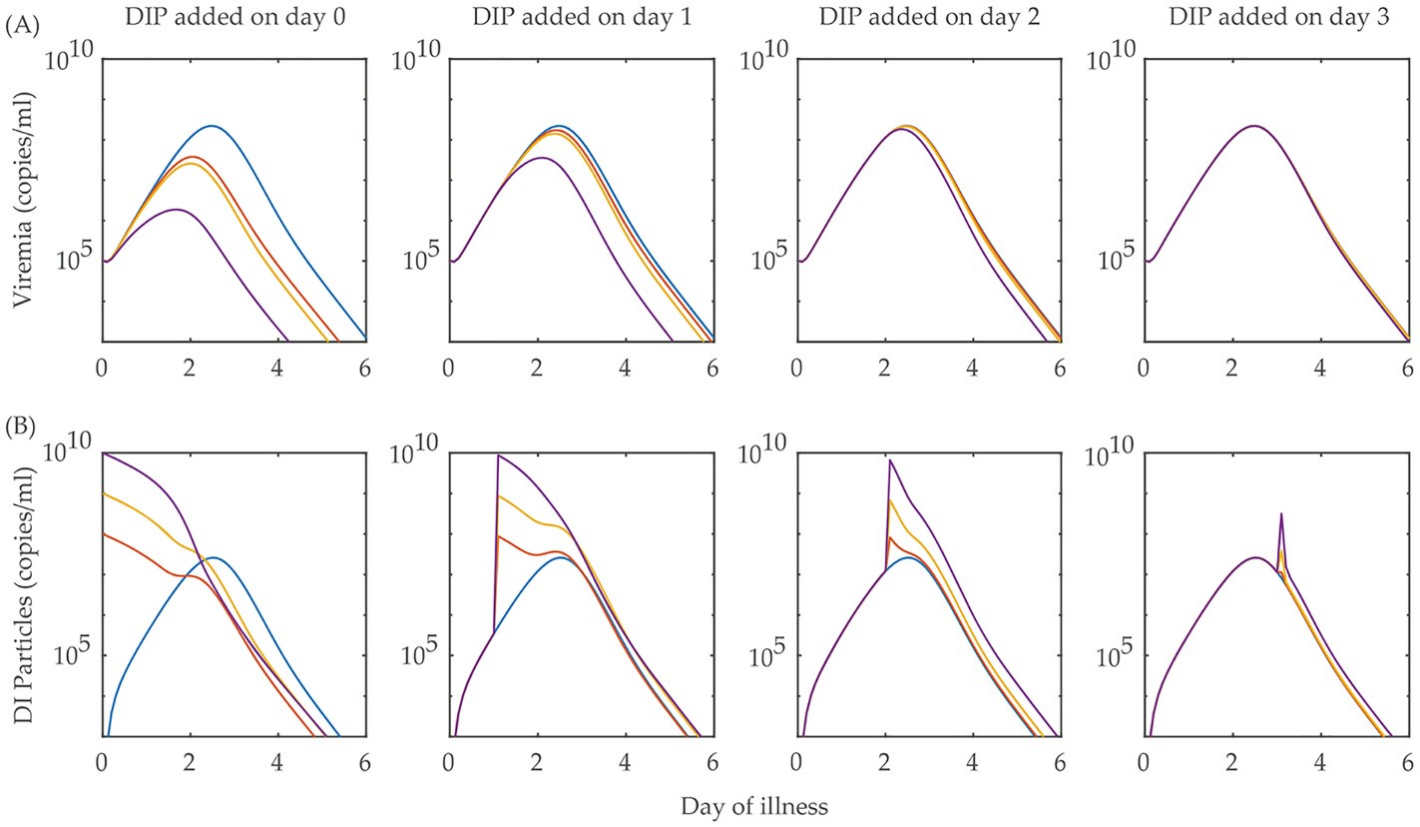
Single time point DI particles (DIP) treatment. Different doses of excess DI particles are added to a within-host dengue viral infection model at different single time points. The effect of the treatment are shown by the dynamics of (A) viraemia and (B) DI particles. The blue lines represent the trajectories of virus and DI particles without applying the treatment of adding DI particles. The lines in red, yellow and purple are the corresponding virus and DI particles dynamics with the addition of 10^8^, 10^9^ and 10^10^ copies of DI particles, respectively on 0, 1, 2 and 3 days of illness. Although a very high dose (10^10^) of DI treatment on day 0 of the illness can reduce the treated viraemia peak by approximately 100 fold and also the duration of illness, the same dose becomes less effective if applied on days 1, 2 and 3 of illness. The other doses of 10^8^ and 10^9^ do not show notable efficiency, even if applied on day 0.

In the first experiment, we observe twelve study sets with three different doses of excess DI particles added on four different days of illness during the increase of the viral load such as, day 0, day 1, day 2 and day 3. Therefore, each study is observed for the entire duration of the fever with a single dose of excess DIPs added on a particular day. Here, we want to mention that in all the models in the four POMs, the initial values of DI particles are kept zero assuming that without any viral infection, DI particles production is not possible. In Fig 6, we present the viraemia and DI particles dynamics for the model. We can observe that a treatment of a very high dose (10^10^) of DI addition before the fever starts, i.e, on day 0, can effectively reduce the viraemia, but it is futile if added after the fever starts. Other lower doses (10^8^ − 10^9^) remain impractical even if they are added on day 0. One might ask why anyone would go for a treatment unless any dengue symptom is observed. This question is answered with the application of optimal bang-bang control treatment of DI particle addition instead of a single time point treatment.

**Fig 7:**
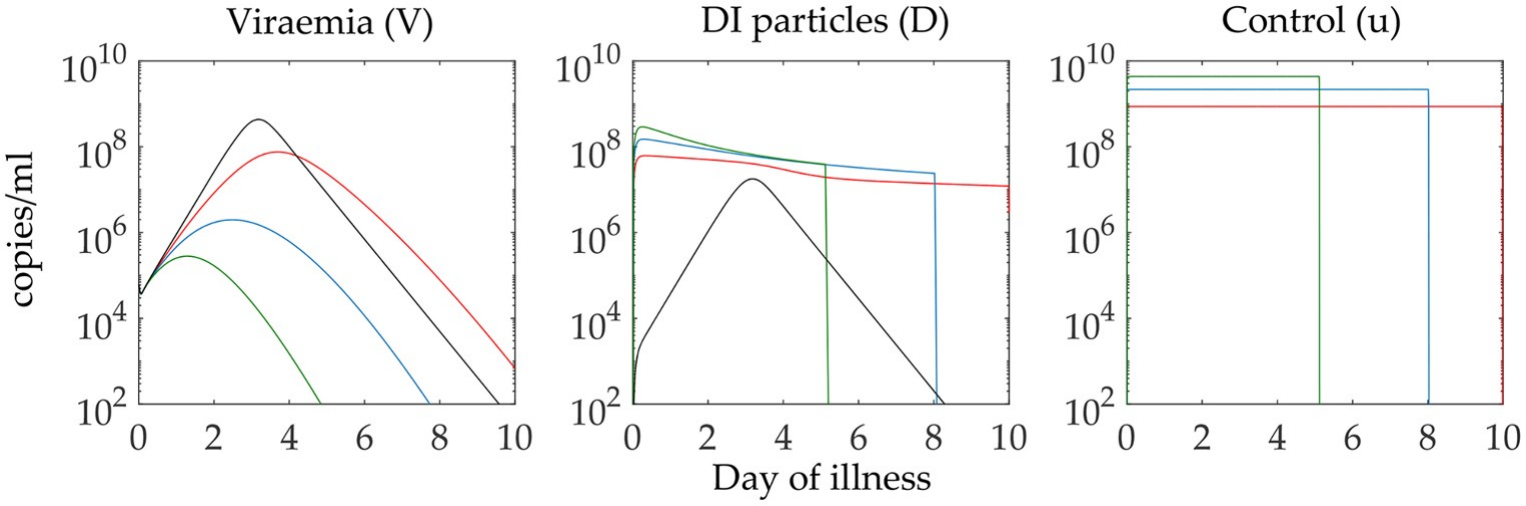
Optimisation of the dose treatment. A very high dose of DI particles treatment can reduce the viraemia peak and clears the viral load within fewer days, but it may not lead to optimal control treatment in terms of the objective function and the expense of the control. For an arbitrary model from the sets of POMs, we observe the effect of different doses (control upper bound) of DI particles addition and solving for the switching time point. The black lines are the trajectories of uncontrolled viraemia and DI particles. The coloured lines are the controlled trajectories of the viraemia and DI particles and the control profiles with different control upper bounds of 1.0 × 10^9^ (red), 2.0 × 10^9^ (blue), and 4.0 × 10^9^ (green) copies of DI particles. We have estimated the efficiency of the control treatment in terms of viraemia reduction later.

In Fig 7 we observe the second experiment, where a course of intervention strategy during early days of illness is successful in reducing the viraemia peak and the duration of virus clearance. In this context it is important to note that increasing the dose strength of DI addition makes the duration of the treatment shorter and the virus is also cleared earlier. However, in terms of the expense of this control treatment (measured by the area under the control curve) with respect to the decrease in viraemia, this may not be optimal. We will discuss on this point later with multiple models from each of the serotype-specific POMs.

### Population of controls

**Fig 8:**
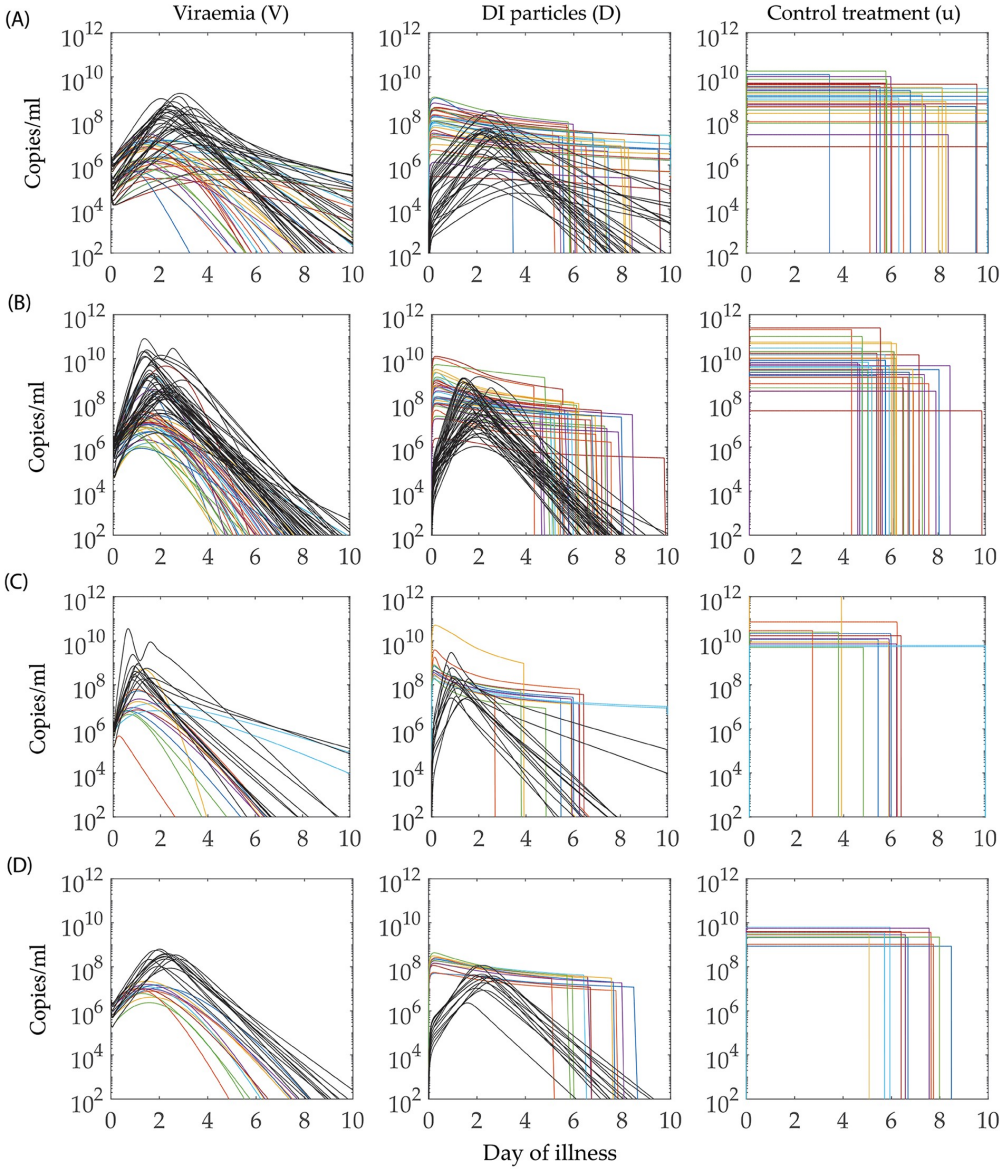
Effect of control treatment on viraemia and defective particles. Representatives of each serotype-specific infected POMs are considered for bang-bang control treatment with addition of excess DI particles as therapeutics. The black lines in the viraemia and DI particles columns are the untreated models from the POMs of (A) DENV-1, (B) DENV-2, (C) DENV-3 and (D) DENV-4, respectively. The coloured lines in the viraemia, DI particles and the control columns show the same models after the treatment with excess DI particles. The colours are used to observe a treated viraemia profile with corresponding treated profile of DI particles and control treatment. Each POMs appears with notable reduction in the viraemia profiles after successful addition of optimal doses of DI particles for optimal time periods.

Once the POMs have been constructed, we approach the problem of predicting the treatment for controlling the fever in the virtual population of dengue patient models. As the total number of calibrated models in the POMs is large (221 for DENV-1, 306 for DENV-2, 93 for DENV-3, and 81 for DENV-4), we randomly choose 15% of the candidate models from each serotype-specific POMs for the control experiment. We sample the models from the POMs with a uniform distribution and obtain 33, 45, 13 and 12 models for DENV-1, DENV-2, DENV-3 and DENV-4, respectively. We could have chosen the best 15% of the best fitted models as the candidates for control experiment, but those do not appear in every domain of the POMs. In Fig 8, we present the viraemia, and DI particle levels before and after applying the control. For DENV-1, the viraemia lasts until day 10 keeping the control on for the whole period in most of the cases, while in case of the other serotypes the control shuts down approximately by day 8. The occurrence of the oscillatory peak in some DENV-2 and DENV-3 models, pushes the control to higher dose although the viraemia cannot last beyond day 5.

If we consider the area under the control curve as the control expense (*A*), then an efficient control must be cost effective. To test the efficiency of the control, we estimate the area under the curve of the viraemia fold reduction (*R*) with respect to the area under the prescribed dose of control curve (*A*). Here the fold reduction (*R*) and control expense (*A*) are defined as

$$R=\frac{\int_{0}^{T}V{\left(t\right)}_{\left(\text{before}\;\text{control}\right)}dt}{\int_{0}^{T}V{\left(t\right)}_{\left(\text{after}\;\text{control}\right)}dt},$$ (11)

$$A=\int_{0}^{T}u\left(t\right)dt,T=10\;\text{days}.$$ (12)

**Fig 9:**
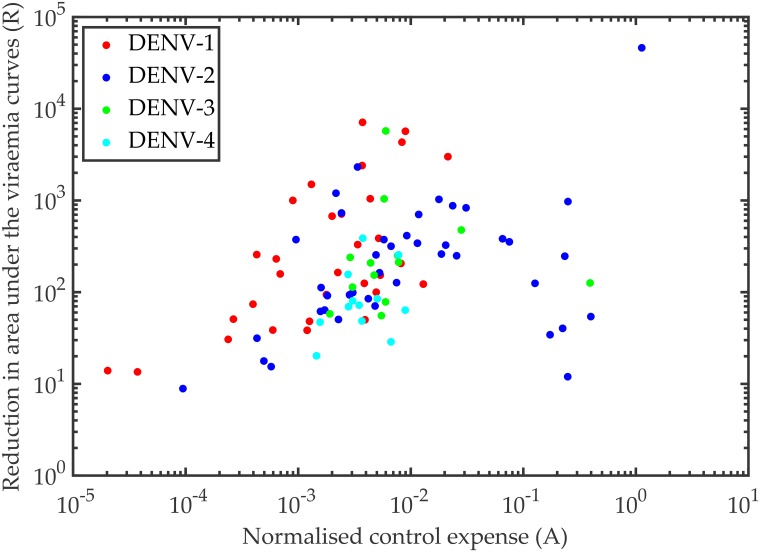
Control efficiency. The efficiency of the intervention strategy using bang-bang control is evaluated here. The control expense (A), which is the area under the control (*u*(*t*)) curve, represent the expense of the treatment for the corresponding patients, who responded with a (R) fold reduction in their corresponding viraemia peak. The (R) fold reduction in the viraemia peak is plotted with respect to the normalised control expense (A) for the four serotypes, DENV-1 (red), DENV-2 (blue), DENV-3 (green), DENV-4 (cyan).

In Fig 9, we show the distribution of the viraemia fold reduction with respect to the control expense for all the four serotypes. Approximate monotonic increments are observed in *R*, with *A* for all the serotypes except DENV-2. For DENV-2, we find two separable clusters; one lies in the same cluster as the other serotypes and the other cluster appears with a completely opposite trend but at higher control expense.

**Fig 10:**
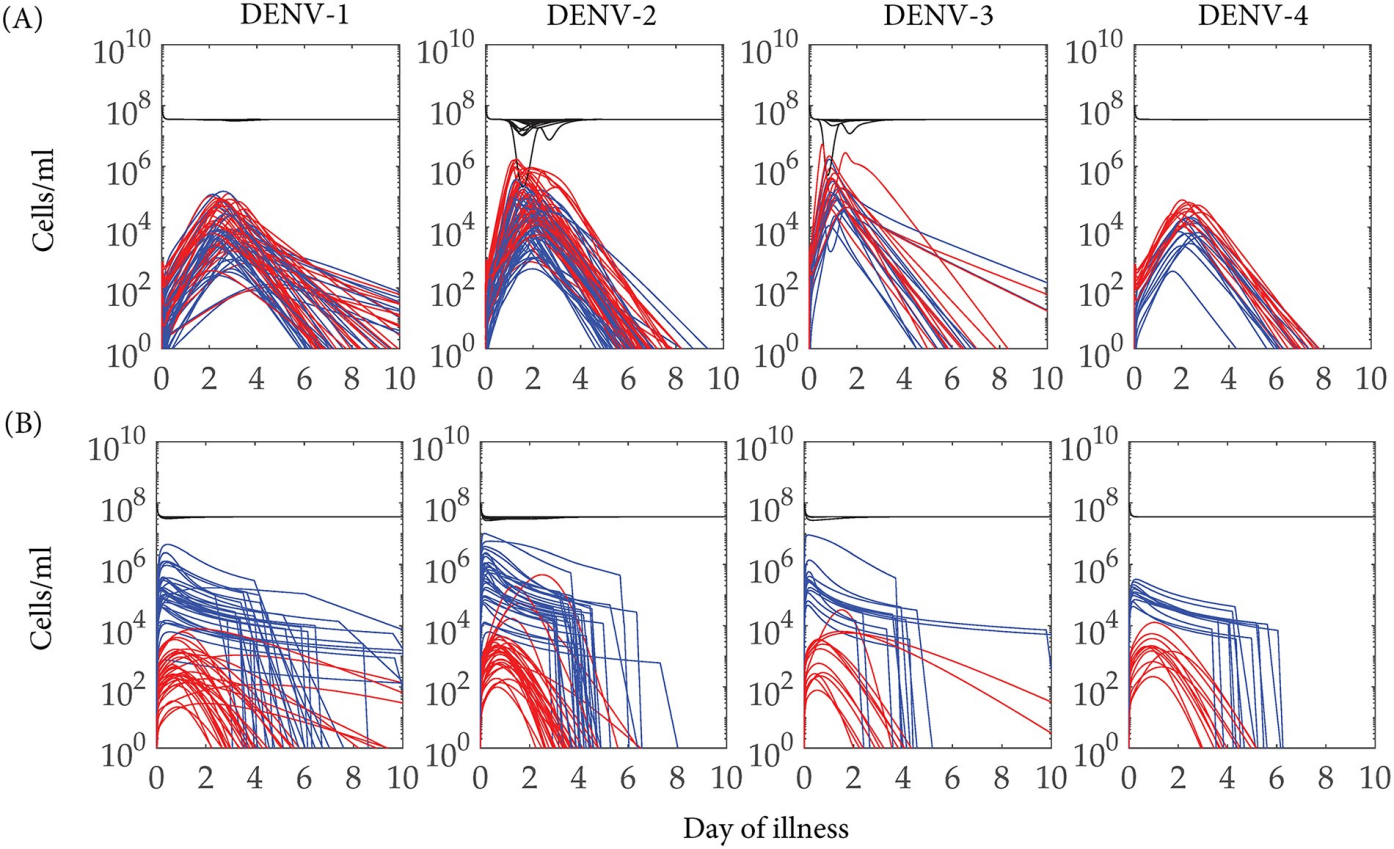
Effect of control treatment on infected cellular dynamics. The dynamics of different cell types are plotted for the candidate models from four serotypes-specific POMs, which are engaged in the control experiment. The uninfected cells (*C*_*U*_) (black), cells infected by DI particles only (*C*_*D*_) (blue) and cells late infected by virus (*C*_V*_) (red) are shown (A) with and (B) without administration of excess DI particles. The *C*_*D*_ cells are cleared from the system after the treatment is over as they cannot replicate DI particles. The control treatment affects the peaks of *C*_V*_ cells as we can see from the rows (A) and (B). The uninfected cell population is not affected greatly with respect to the infected cells population.

The infected cellular dynamics also shows remarkable changes after the application of excess DI particles in the host system (Fig 10). The general trend before and after applying the control is observed in the *C*_*D*_ cells, which is similar to that of the DI particles, as the DI particles are the major reason to generate the pool of *C*_*D*_ cells. A similar relation is observed between the *C*_V*_ cells with the viraemia profile as only *C*_V*_ cells release potential virus into the body fluid. Interestingly, the application of the excess DI particles starts inhibiting both the virus and the *C*_V*_ cells. The population of *C*_*D*_ cells are produced from *C*_*U*_ cells upon being infected by *D* and *C*_*VD*_ cells produce *D*. As a result, the pool of the DI particles drops sharply as soon as the control shuts down and the consequences are reflected in the numbers of *C*_*D*_ and *C*_*VD*_ cells.

## Discussion

The two prime interests of this paper are to capture the inherent variability in dengue-infected patient data through a within-host model and predict efficient intervention to control dengue fever via administration of excess defective interfering particles (DI particles). We present the method of population of models (POMs) to execute the first goal and a population of optimal bang-bang control settings for the second aim. We show that the POMs not only capture the biomarker dataset but also provides the range of variability for each cell-virus interaction and its association with the biomarker kinetics in population and individual levels. A sub-population of the calibrated POMs are used with bang-bang control to reduce the viraemias in significant orders. In that case, the fever cannot reach the state of severe dengue and the DI particles do not stop replicating. As per our findings, the antiviral property of the DI particles appears as a potential intervention strategy to attenuate the patient viraemia significantly.

We construct four serotype-specific populations of within-host models for dengue against the variability in the biomarker levels in blood samples of the admitted patients as reported [8]. The four sets of POMs explore a range of patient-specific parameters, those in different combinations, produce four populations of feasible dengue models within the range of the experimental data. The calibration of the POMs helps us to discriminate and classify among the serotypes and inter-patient variability through parameter variability and sensitivity. The aim of this methodology is not to look at the dynamics of isolated models in the population as any single model does not represent an individual. The aim is to incorporate variability in the same model and observe the whole population of patients with similar symptoms.

Variability appears in the population of the viraemia load and the corresponding antibody response due to the differences in the patient-specific parameters. One of the crucial factors that drives this variability is the incubation period for an individual model. We want to mention that we trace the variability of incubation periods of an individual model in terms of the variability in viral load on day 0 of illness (*V*_0_) and that efficiently fits with the calibration process. The dynamics of the viraemia (*V*) is directly dependent on the rate of infected cell death (*δ*), maturation rate of *C*_*V*_ cells (*π*_1_), rate of virus production (*π*_2_) for release after maturation of the infected *C*_*V*_ to *C*_V*_ and on the antibody response (*Z*) for clearance. Indirectly, the rate of infection (*k*) also drives the viraemia. Amongst these parameters, *δ* is in strong positive correlation with *V*, *Z* and *D* and that gradually leads to a flip as the viraemia dies with the days of illness, but *π*_1_ is weakly correlated all the time. The variability of highly correlated parameters stay within a narrow range and calibrate tightly with the biomarker data, but weakly correlated parameters spread over wide ranges to generate models with similar behavior (Figs 4 and 5). Quantitatively, the strength of correlation of the parameters with the clinical biomarker data is an estimate of the sensitivity of the model parameters onto the serotype-specific POMs. Here, variability in the highly sensitive mature infected cell (*C*_V*_) death rate (*δ*) has a more significant role than the comparatively lower sensitive maturation rate (*π*_1_) of *C*_*V*_ cells in the variability of the virus clearance and duration of dengue fever. The dynamics of *C*_V*_ in Eq 1 informs the possible structure of the objective function for an efficient optimal control based on the comparative parameter sensitivity. In the present optimisation problem, we minimise the early infected cells (*C*_*V*_) with the plasma viral load (*V*), not the late infected cells (*C*_V*_) (see Eq 9).

In the Ben-Shachar *et. al*. [48] statistical model, the populations of infected patients have been classified according to the disease severity across the serotypes and the variability in their immune responses. Although this study is more concerned with the immune response, they predicted the relation among virus replication rates with the timing of the viraemia peaks over the days of illness. Our POMs results show consistency with their observations when we demonstrate the variability for different parameters. DENV-1 and DENV-4 reach the viraemia peaks after the symptom onset, while the peaks appear before the onset of the symptoms in case of DENV-2 and DENV-3 and it depends on the degree of infection (Fig 2). However, the few relatively high peak heights in viraemia data for DENV-1 cannot be captured in our model.

Among the reported infections of the hospitalised patients in our model, most of the DENV-1 infected patients have primary infection while the majority of the patients with the other serotypes are reported as secondary infection. A careful observation of the POMs of the viraemia profiles enables us to find the growth rate of the viraemia for most of the models, with the DENV-2 and DENV-3 POMs growing faster than the others. We explain this rapid growth in terms of the antibody dependent enhancement (ADE) that only occurs in secondary infection [6]. In case of primary infection, the immune response is triggered very slowly and the viraemia is almost cleared when the response level is significant. On the other hand, the same response for the secondary infection is very rapid and prominent.

In the articles of Clapham *et. al*., two different within-host models for dengue infection have been presented for DENV-1 and DENV-2. These authors studied variability in the rate of infection (*k*) only and that was used to discriminate between the ranges of viraemia loads [20]. Later they have calibrated two improved models with direct and indirect effects of the antibody response through free virus neutralisation and infected cell death [19]. In the present article, we keep the rate of infection (*k*) and rate of antibody-mediated virus neutralisation (*ϵ*) constant for each serotype and included the immune cell-mediated antibody production, which is triggered by both the free virus and free defective particles. The variability in the antibody production is captured by *η*_1_ and *η*_2_ and their contributions are reflected in the construction of the POMs. The greater the proliferation (*η*_1_) rate varies, the more the antibody plateau widens (Figs 2 and 4). Notably, in the case of DENV-4, the spread for both of *η*_1_ and *η*_2_ are narrow. The strong negative correlation of *η*_1_ with the viraemia does not appear to be significant in comparison with the case of the DI particles and immune response. This may explain the delay in the triggered antibody response generated by *D*. In principle, the antibody response is activated after the virus population (standard or defective) has reached its peak and the production of DI particles is marginally delayed. This observation suggests a cue to investigate the different time-scales of the virus proliferation and activation of the immune response.

Another significant outcome of such a population level modeling approach is in the evaluation of the dose and duration of an intervention strategy for dengue fever. We use a bang-bang control approach to model adding excess DI particles into the dengue-infected host system in order to reduce the plasma viral burden and the fever. Note that the optimal control gives an optimal dose in a mathematical setting. Of course this may not be optimal in a real life setting due to other factors that are beyond the scope of the model to represent. This of course is always the case when modelling is done. Previously, Rodrigues *et. al*. showed optimal control for dengue using vaccination compartment inside an epidemic viewpoint [18]. But intervening individual human host models within a population has not been observed yet. Furthermore, the naturally occurring defective interfering particles have not been utilised in dengue control before.

We perform the control experiment on a randomly chosen 15 per cent of models from the calibrated population of models for each serotype. In Fig 8, the population of controlled models (cPOMs) and population of controls (POCs) profiles for the four serotypes are presented with the uncontrolled POMs. As the replication of the DI genomes depends on the replicative machinery synthesised by the standard viral genomes, the excess DI particles are rapidly cleared out of the host system as soon as the control shuts down and viraemia is cleared. We ensure the amplitude of the control, i.e., addition of excess DI particles, to be equivalent to the level of viraemia peak during the controls, otherwise the amount of the DI particles are not sufficient to reduce the viraemia peak. Our aim is to keep the viral load approximately below 10^8^ but for DENV-2 and DENV-3 it is difficult to achieve that even after applying 10^11^ DI particles. The reason behind this is the higher rates of virus replication (*β* and *π*_2_) in DENV-2 and DENV-3, as mentioned before. In the cases of DENV-1 and DENV-4, as soon as the DI particles start boosting, the viral load drops quickly, as the DI particles interfere in the virus replication. Very tiny persistent oscillations in the case of DENV-2 and DENV-3 in all the cell types and viraemia also validates the same conclusions.

To examine the efficiency of the control experiment, we refer to the scatter plot in Fig 9 for the measured control expense (*A*) and the corresponding reduction in viraemia (*R*). For DENV-1, DENV-3 and DENV-4, most of the models are in the left half of the figure (i.e., *A* ≤ 10^3^) while DENV-2 has many more models in the high *A* domain (i.e., *A* ≥ 10^3^). In most of the cases for DENV-1, the reduction (*R*) is higher than the other serotypes at low expense of control (*A*) and that makes the control for DENV-1 the most efficient. The present model predicts that large numbers of DI particles would be administered to DENV patients to have any effect on viraemia as patients only become symptomatic and seek medical assistance at the time of peak viraemia or soon after. The model also assumes that DI particles and wild type viruses are of equal fitness when competing for replicative machinery within the host cell. If, however, DI particles are interfering with replication of wild type viruses by enhancing production of interferon or some other mediator, then a single DI particle/genome may elicit a response in the host cell that interferes with the replication of large number of wild type viruses. In addition, there exists no specific metric that may provide room to define the efficiency of the DI particles. A distribution of DI genomes with variability in their competitions with the virus RNAs for the replication and packaging can be modelled to predict the efficiency of the DI particles through successive passages. Although, existing models and experiments with DI particles assume the efficiency of the DI particles is inversely proportional to their nucleotide lengths [23], just the nucleotide lengths cannot decide on DI particle efficiency. Shorter genome length may help DI particles in faster replication, but deletion mutations only at the genes of non-structural proteins can increase the DI particles efficiency, which is a random event. Hence, a single cell stochastic model with distribution of DI particles and their evolutionary aspects may open a new avenue to explore DI particle efficiency.

Despite the availability of real clinical data for the admitted patients and experimental success, the intra-host dengue virus dynamics is not explored well. As a consequence, the virus transmission dynamics to mosquitoes is not clear. This paper explores the variability regime of the intra-host DENV dynamics across a population of patients for the four DENV serotypes. These POMs are able to predict the effective roles of the virus replication and subsequent immune response to determine the within-host viraemia characteristics. For the same patients population, a human to mosquito transmission model is underway. Those results may explore the quantitative analysis of infected patients turned into infectious and their infectiousness in terms of the transmission. Addition of minimal amount of defective particles leads to significant reduction in the viraemia characteristics reflecting the potential anti-viral property to be manifested in dengue control.

## Supporting information

S1 Fig An arbitrarily chosen model from the POM is simulated with and without the ‘death’ terms for the *C*_*D*_ (*δC*_*D*_) and *C*_*V*_ (*δC*_*V*_) cells in Eq 1. The effect of the ‘death’ terms on the model are shown by the viraemia (*V*), defective interfering particles (*D*), and two early infected (*C*_*D*_ and *C*_*V*_) cells levels. The black lines represent the model without the ‘death’ terms, while the red lines show the model dynamics with the ‘death’ terms. These results reflect that inclusion of the ‘death’ terms cannot contribute significantly to the model dynamics except the stability of the *C*_*D*_ cells.
(TIF)
