# Supplementary figures and images for: A population of bang-bang switches of defective interfering particles makes within-host dynamics of dengue virus controllable
Source: PLoS Comput Biol. 2019 Nov 11;15(11):e1006668. doi: 10.1371/journal.pcbi.1006668 (PMC6872170; doi:10.1371/journal.pcbi.1006668)

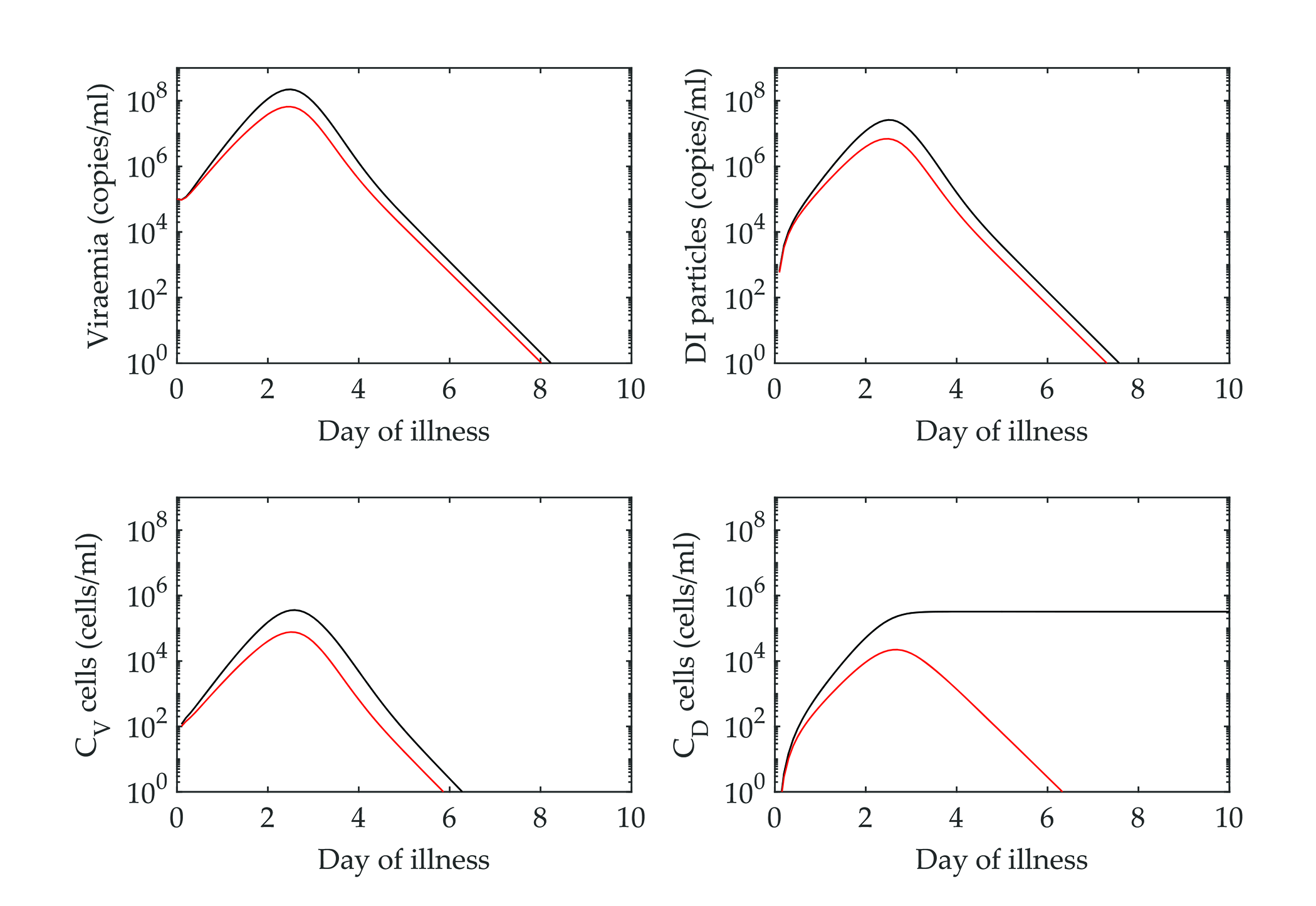

Supplement: S1 Fig — The effect of the ‘death’ terms on the model are shown by the viraemia (V), defective interfering particles (D), and two early infected (CD and CV) cells levels. The black lines represent the model without the ‘death’ terms, while the red lines show the model dynamics with the ‘death’ terms. These results reflect that inclusion of the ‘death’ terms cannot contribute significantly to the model dynamics except the stability of the CD cells. (TIF) [file pcbi.1006668.s001.tif]
